# The utility of PET imaging in depression

**DOI:** 10.3389/fpsyt.2024.1322118

**Published:** 2024-04-22

**Authors:** Shashi B. Singh, Atit Tiwari, Maanya R. Katta, Riju Kafle, Cyrus Ayubcha, Krishna H. Patel, Yash Bhattarai, Thomas J. Werner, Abass Alavi, Mona-Elisabeth Revheim

**Affiliations:** ^1^Department of Radiology, Stanford University School of Medicine, Stanford, CA, United States; ^2^BP Koirala Institute of Health Sciences, Dharan, Nepal; ^3^University of La Verne, La Verne, CA, United States; ^4^Rhythm Neuropsychiatry Hospital and Research Center Pvt. Ltd, Lalitpur, Nepal; ^5^Harvard Medical School, Boston, MA, United States; ^6^Department of Epidemiology, Harvard T.H. Chan School of Public Health, Boston, MA, United States; ^7^Icahn School of Medicine at Mount Sinai, New York City, NY, United States; ^8^Case Western Reserve University/The MetroHealth System, Cleveland, OH, United States; ^9^Department of Radiology, Hospital of the University of Pennsylvania, Philadelphia, PA, United States; ^10^The Intervention Center, Division of Technology and Innovation, Oslo University Hospital, Oslo, Norway; ^11^Institute of Clinical Medicine, Faculty of Medicine, University of Oslo, Oslo, Norway

**Keywords:** depression, PET, antidepressant, FDG, serotonin, amyloid, electroconvulsive therapy (ECT), deep brain stimulation (DBS)

## Abstract

This educational review article aims to discuss growing evidence from PET studies in the diagnosis and treatment of depression. PET has been used in depression to explore the neurotransmitters involved, the alterations in neuroreceptors, non-neuroreceptor targets (e.g., microglia and astrocytes), the severity and duration of the disease, the pharmacodynamics of various antidepressants, and neurobiological mechanisms of non-pharmacological therapies like psychotherapy, electroconvulsive therapy, and deep brain stimulation therapy, by showing changes in brain metabolism and receptor and non-receptor targets. Studies have revealed alterations in neurotransmitter systems such as serotonin, dopamine, GABA, and glutamate, which are linked to the pathophysiology of depression. Overall, PET imaging has furthered the neurobiological understanding of depression. Despite these advancements, PET findings have not yet led to significant changes in evidence-based practices. Addressing the reasons behind inconsistencies in PET imaging results, conducting large sample size studies with a more standardized methodological approach, and investigating further the genetic and neurobiological aspects of depression may better leverage PET imaging in future studies.

## Introduction

According to the WHO, depression affects approximately 5% of the world’s population and is a major contributor to disability and global health challenges (1). Based on estimates from the National Institute of Mental Health, 21 million adults in the United States experienced one or more depressive episodes in 2021, with adult females (10.3%) experiencing higher rates of depressive episodes than males (6.2%) (2). Depression is characterized by lasting sadness, low mood, and reduced pleasure in previously pleasurable activities. Other clinical symptoms include sleep and appetite disturbances, poor concentration, and fatigue. Consequences of depression can be long-lasting or recurrent and can significantly impact patients’ ability to function, often leading to somatic symptoms and worsened physical health (1). Depression is precipitated and exacerbated by complex social, psychological, and biological interactions. For instance, unemployment, grief or mourning, and traumatic experiences all increase vulnerability to depression. Depression is currently diagnosed based on criteria set by the Diagnostic and Statistical Manual of Mental Disorders-5 (DSM-5) or International Classification of Diseases-11 (ICD-11). Depression has several treatment options of increasing intensity and support. Selective-serotonin reuptake inhibitors (SSRIs) are used most frequently in the first-line setting, but serotonin-norepinephrine reuptake inhibitors (SNRIs) and tricyclic antidepressants (TCAs) are used in second and third-line cases depending on the severity and pattern of depressive episodes as well as other comorbid conditions like chronic pain (1). In addition to pharmacological therapies, psychological therapies (such as cognitive behavioral therapy, interpersonal psychotherapy, and behavioral activation therapy) have been used to various degrees in treating depression (1).

Advances in positron emission tomography (PET) imaging techniques and radiotracers have elucidated mechanisms of disease that increase understanding of depression and provide a foundation for translational research and drug development. For example, PET allows for advanced imaging of glucose metabolism and examination of complex biological activity in healthy and pathological states through the creation of three-dimensional (3D) maps using positron-emitting radiopharmaceuticals like [^18^F]fluorodeoxyglucose [(^18^F)FDG] (3–5). In particular, PET has shown that abnormalities of 5-HT_1A_ and 5-HT_1B_ receptors, subtypes of serotonin receptors located in presynaptic and postsynaptic regions, are hallmarks of affective disorders like depression (6–9). In this educational primer, we emphasize the applications of PET in depression. We begin with a brief overview of PET, followed by a discussion of the primary clinical applications of PET and its radiotracers in the diagnosis and management of depression. We conclude with a discussion of how PET imaging can contribute to future depression research.

## Positron emission tomography

### Principle and mechanism

PET is an imaging procedure that allows for 3D mapping of administered positron-emitting radiopharmaceuticals and facilitates the study of biological activity (3, 10). Radioisotopes used in PET reach stable configurations by the emission of positrons. The positrons emitted from radioisotopes travel a few millimeters through surrounding tissues, which causes a significant loss in kinetic energy. Then, the slowed positrons interact with electrons to generate two 511 keV γ-rays, which travel approximately but not completely in opposite directions. PET scanning comprehensively detects millions of coincidental events between these rays to display the concentration and spatial distribution of positron emitters within the patient (11, 12). The distance traveled by the positron before annihilation (positron range) varies depending on the energy of the positron and the density of the surrounding tissue. Higher energy positrons travel further, leading to a larger positron range, which can reduce the spatial resolution of the PET image.

### Radiotracers

The first step in developing the radioactive tracers used for PET imaging is understanding the biological target, most notably its expression level, brain distribution pattern, and their interactions with radiotracers. It is paramount that the radioligands (injected in tracer doses) used in PET imaging and labeling are stable and have high selectivity, specificity, and affinity for a target with minimal off-target binding. In brain PET, they must also be able to pass through the blood-brain barrier to reach the target. The most commonly used radioisotopes for labeling radiopharmaceuticals are [^11^C] and [^18^F] (13). PET is most commonly used in clinical practice with the radiotracer [^18^F]FDG, an analog of glucose (14). Although [^18^F]FDG PET is used clinically in oncology, none of PET is used in clinical psychiatric applications currently. In research settings, [^18^F]FDG PET sometimes involves blood sampling and full quantification, but most often [^18^F]FDG PET does not involve blood sampling with the use of a semi quantitative approach (15). The development of radiotracers has aided in better understanding the various neurobiological mechanisms, neural pathologies, and pharmacodynamics of drugs used to treat multiple neuropsychiatric disorders, including depression. [^11^C]DASB and [^11^C]MADAM(N,N-dimethyl-2-(2-amino-4-methylphenyl-thio)benzylamine) are well-known PET radioligands for serotonin transporters, given their high specificity and selectivity for binding sites; this in turn allows for reliable estimation of the serotonin transporters (16). Similarly, [^18^F]FMeNER-D2 (S,S)-2-(α-(2-[^18^F]fluoro[2H2]methoxyphenoxy) benzyl)morpholine) has been used as a radioligand for norepinephrine-based PET studies (17, 18). **Table 1** lists a sample of currently available PET radiotracers that have been studied in depression, gathered from references included in this article. Importantly, although PET radiotracers used in depression have enhanced our understanding of its pathophysiology and treatment response, none of them have been able to make it to clinical practice yet.

**Table 1:** Examples of PET radiotracers that have been studied in depression, gathered from references included in this article.

Target	Radiotracers
Serotonin	[^11^C]DASB, [^11^C]MADAM (specific to serotonin transporter) [^11^C]WAY 100635 (specific to serotonin _1A_ receptor), [^11^C]AZ10419369 (selective to serotonin _1B_ receptor)
Dopamine	[^18^F]FE-PE2I (binds to dopamine transporter) [^11^C]raclopride (binds to D2 dopamine receptor) [^18^F]fallypride, [^11^C]FLB 457 (binds to D2 and D3 dopamine receptors) [^18^F]FDOPA (taken up by dopaminergic neurons and converted into fluorodopamine, which then accumulates in presynaptic dopamine storage vesicles)
Norepinephrine	[^18^F]FMeNER-D2 (binds to norepinephrine transporter)
Gamma-Aminobutyric Acid	[^11^C]Flumazenil (binds to GABA-A benzodiazepine receptor)
Opioid	[^11^C]carfentanil (binds to Mu-opioid receptors)
Tau	[^18^F]flortaucipir (binds to tau protein and detect its deposits)
Amyloid	[^18^F]florbetapir, [^11^C]-Pittsburgh Compound B (binds to amyloid plaques)
Glutamate	[^11^C]ABP688 (binds to Metabotropic glutamate receptor subtype 5 i.e. mGluR5 receptor)
Translocator protein	[^18^F]FEPPA (binds to 18-kDa Translocator protein found predominantly on the mitochondrial membrane of microglial cells, used to assess neuroinflammation)

### Limitations of PET

Although PET is a promising modality for the diagnosis, management, and monitoring of neuropsychiatric disorders, it has several limitations, including limited accessibility (especially in developing nations), temporally lengthy examination, high operating costs, physical constraints, and difficulty manufacturing radiotracers (19). Due to the utilization of radioactive tracers, PET imaging has a concomitant potential for radiation exposure. On average, a brain [^18^F]FDG PET scan entails exposure of approximately 5-7 milliSievert (a measure of radiation exposure), contingent upon the specific imaging protocol (20). Moreover, the need for further improved sensitivity and anatomical resolution (∼5 mm) remains (21, 22).

When [^18^F]FDG is used with PET to image regional cerebral glucose metabolism in neuropsychiatric diseases, physiologically high [^18^F]FDG uptake in the brain poses a challenge for appreciating findings (23, 24). Approximately 95% of the energy consumption required for brain function is provided by glucose metabolism. The cerebral glucose metabolism is closely linked to neuronal activity, and changes in neuronal activity induced by diseases are reflected as an alteration in glucose metabolism. The high glucose metabolism may be challenging in all brain studies with possible alteration of the metabolism, for example, due to psychotropic drugs or other interventions. Furthermore, the blood glucose level significantly affects the [^18^F]FDG uptake of the brain when hyperglycemia is present. There is an increased competition between elevated plasma glucose and [^18^F]FDG during hyperglycemia, and the uptake of [^18^F]FDG decreases in such situations. Thus, due to the high physiological uptake of [^18^F]FDG in normal brain gray matter, moderate to minor alterations in [^18^F]FDG PET may be challenging to detect (23, 24). Therefore, a standardized acquisition protocol is recommended to improve the comparability between subsequent scans or among different patients.

PET imaging may also be uncomfortable for patients with neuropsychiatric disorders or physical disabilities, as stillness is required during imaging. Further developed individualized protocols for patients with these needs are necessary to reduce scan time and address discomfort associated with the procedure.

## The utility of PET in the diagnosis of depression

In patients with depression, PET imaging generally reveals generalized malfunction shown as reductions in cerebral blood flow and metabolism (25, 26). [^18^F]FDG PET is useful in the study of depression due to its ability to measure regional cerebral glucose metabolism. Glucose metabolism can demonstrate brain activity, while changes in regional metabolism can provide insights into the underlying neural mechanisms associated with depression (27, 28). Oxygen-15 H_2_O [(^15^O)-water] PET is another useful technique in depression due to its ability to measure regional cerebral blood flow, providing information about blood perfusion and neuronal activity. Changes in blood flow are associated with alterations in neural activity, making [^15^O]-water PET a valuable tool for understanding the vascular aspects of depression (29, 30). In depression, [^18^F]FDG PET measures long-term changes in glucose metabolism, reflecting longer-term changes in neural activity, while [^15^O]-water PET specifically assesses cerebral blood flow, providing a dynamic snapshot of immediate effects on blood perfusion in brain regions associated with mood regulation. The physiological specificity of [^15^O]-water PET complements the metabolic information from [^18^F]FDG PET, offering a better understanding of the neural and vascular factors in depression. Changes in glucose metabolism patterns and vascular functions may also indicate treatment response or help identify potential biomarkers for treatment outcomes. However, discrepancies may exist between papers that study [^18^F]FDG or [^15^O]-water PET in depression; the discrepancies are usually due to methodological differences, patient heterogeneity, or sample size considerations (27–30). An early PET study in patients with depression used [^15^O]-water to demonstrate decreased blood flow in both the left dorsolateral prefrontal cortex and left anterior cingulate gyrus (31). A separate but similar PET study conducted with [^15^O]-water showed decreased cerebral blood flow in the left anterior medial prefrontal cortex and increased cerebral blood flow in the cerebellar vermis of patients with comorbid cognitive impairment and depression (32).

Similarly, [^18^F]FDG PET has shown distinct utility in depressed patients by demonstrating uniquely lower glucose metabolism of glucose in cortical, subcortical, and cerebellar regions as well as the frontal and limbic systems (33, 34). Additional [^18^F]FDG PET studies showed globally lowered brain metabolism in bipolar patients in a depressive episode (27, 28).

Studies have also been conducted to identify the role of tau and amyloid proteins in the pathophysiology of depression. Tau and amyloid pathology are hallmarks of certain neurodegenerative disorders notably Alzheimer’s Disease. These proteins are less directly implicated in the commonly proposed pathophysiology of depression. Nevertheless, clinical co-occurrence of depression and dementia or neurodegenerative disease remains high where those with Alzheimer’s disease often experience mood changes (29), including symptoms of depression, whereas patients with depression also have cognitive difficulties (30). Pathologically, the accumulation of beta-amyloid plaques and tau tangles in Alzheimer’s disease are known to disrupt neurotransmitter systems, particularly serotonin and norepinephrine, linking the neurobiological changes to mood alterations observed in depression (35). A PET study by Gonzales et al. using tracers [^11^C]-Pittsburgh Compound B ([^11^C]PiB) and [^18^F]flortaucipir tau showed that midlife depression and tau-PET uptake in the amygdala and entorhinal cortex were positively associated (36). In another PET radiotracer study using [^18^F]flortaucipir (AV1451) for tau and [^18^F]florbetapir (AV45) for amyloid, tau uptake was again linked to depression in patients with normal cognition (37). However, there was no association between depression and amyloid in this study (37). Notably, the evidence derived from PET studies around beta-amyloid (Aβ) accumulation in late-life depression is mixed. While studies have shown that late-life depression is not associated with higher cortical Aβ accumulation (38–40), one PET study by Smith et al. using [^11^C]PiB found that patients with late-life depression had more Aβ accumulation in the left parietal cortex in comparison to patients in the control group. Here, the degree of Aβ deposition in the left parietal cortex was linked to more severe depressive symptoms and impairment of visual-spatial memory as well as increased likelihood of unsuccessful clinical improvement after treatment with SSRIs (41).

**Figure 1:**
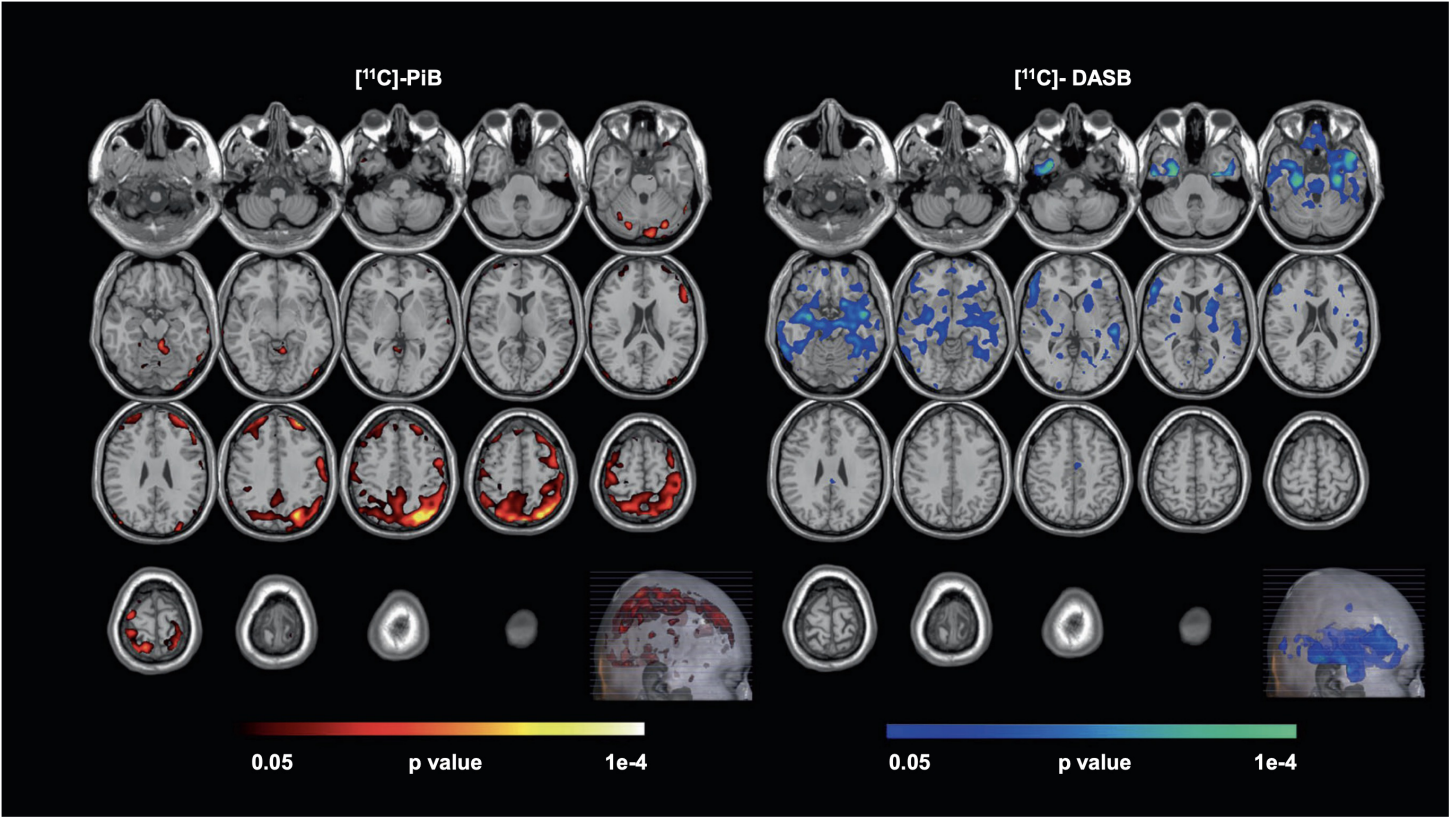
Voxel-wise, statistical parametric mapping unimodal analysis for [^11^C]-PiB and [^11^C]-DASB. Voxel-wise, statistical parametric mapping and separate unimodal analysis for [^11^C]-PiB and [^11^C]-DASB showed higher beta-amyloid (left, hot-colored areas) and lower serotonin transporter availability (right, cool-colored areas) observed in Late-Life Depression (LLD) patients when compared to normal controls. Here, the Monte-Carlo Simulation Method is used to address the issue of multiple comparison correction in this study [with permission from reference (42)].

Late-life depression also increases patients’ future risk of cognitive decline. A recent PET investigation among 20 unmedicated late-life depressive patients and 20 controls was conducted using tracers [^11^C]PiB (for Aβ) and [^11^C]DASB (for serotonin transporter). The parametric PET data was processed using a multi-modal partial least squares (mmPLS) algorithm. Patients with late-life depression were distinguished from healthy controls with a spatial covariance pattern showing higher levels of Aβ in the temporal, parietal, and occipital cortices and lower levels of 5-HTT in the putamen, thalamus, amygdala, hippocampus, and raphe nuclei. Stronger expressions of the spatial covariance pattern, linking elevated beta-amyloid and reduced serotonin transporter levels, directly correlated with increased severity of depressive symptoms (**Figure 1**), suggesting a potential neurobiological mechanism underlying late-life depression (42). Although the study suggests that in late-life depression patients there is an inverse relationship between beta-amyloid and serotonin transporter levels, the exact reason for this inverse relationship and its implications for younger-onset depression is not clear. The inverse relation between beta-amyloid and serotonin transporter levels is hypothesized to result from complex interactions between neurochemical and neurodegenerative mechanisms common to depression and dementia, but further investigation is needed particularly in the context of younger onset depression. Regardless, this unique pattern might serve as a biological marker for antidepressant treatment response and/or cognitive decline in late-life depression patients.

A meta-analysis explored 5-HT_1A_ density and its binding in individuals with depression compared to healthy controls. There was a significant decrease in 5-HT_1A_ density observed in the mesiotemporal cortex of depressed patients. In addition, smaller reductions in 5-HT_1A_ receptor binding were noted in the hippocampus, insular cortex, raphe nucleus, occipital cortex, and anterior cingulate cortex in the depressed group (43). However, other PET studies using different quantification techniques have produced conflicting findings in patients with depression (44). For example, unlike the above meta-analysis, where there was a decrease in 5-HT_1A_ density in depression, another study reported increased 5-HT_1A_ density in depression (45). Nevertheless, when full quantification is used with PET, 5-HT_1A_ density has been found to be higher in depressed patients; this fact highlights the critical nature of fully quantitative PET in research. The higher 5-HT_1A_ density in depression observed during full quantification is possibly due to compensatory upregulation of 5-HT_1A_ receptor expression in response to serotonin dysregulation, a phenomenon commonly observed in depression (46). On the other hand, in terms of 5-HT_1A_ transporter, a PET study using [^11^C]-DASB tracer revealed patients with major depression had decreased binding of the serotonin transporter in the frontal cortex, anterior cingulate cortex, brainstem, caudate-putamen, and thalamus (47).

Emerging evidence from recent studies suggests that changes in excitatory neurotransmission (i.e., glutamate) play a role in the pathophysiology of depression (48, 49). Using PET scans with [^11^C]ABP688 tracer, Deschwanden et al. studied the binding of metabotropic glutamate receptor 5 (mGluR5) in 11 unmedicated patients with major depressive disorder vs. 11 healthy controls (48). The [^11^C]ABP688 PET scans showed that those with depression had lower mGluR5 binding in various brain regions, including the prefrontal and cingulate cortex, insula, thalamus, and hippocampus. Moreover, the severity of depression was associated with reduced mGluR5 binding in the hippocampus. Thus, [^11^C]ABP688 PET suggested changes in excitatory neurotransmission may be involved in the development of major depressive disorder. These findings were supported by western blot analysis of postmortem brain samples of 15 deceased individuals who had depression and 15 who did not. The analysis found lower mGluR5 protein expression in the prefrontal cortex of the depressed group (48). Similarly, Kim et al. conducted another study using [^11^C]ABP688 PET on 16 patients with major depression who had not taken any psychotropic drugs and had no other mental health conditions, comparing them with 15 healthy individuals (49). Their analysis showed that the depressed patients had significantly reduced mGluR5 availability, especially in the prefrontal cortex and other brain regions, such as the temporal and parietal cortices, further supporting the glutamatergic hypothesis of depression. Additionally, using resting-state functional MRI, the authors also found notable differences in brain connectivity, with the depressed group showing less negative connectivity out of the inferior cortical areas than that seen in controls (49). Note that while the aforementioned studies are not an exhaustive compendium of all evidence regarding mGluR5 binding, they collectively represent the most recent, rigorous data, which was also collected through more refined methods and advanced technology than data from less recent studies.

Other neurotransmitters have also been found to be associated with clinical manifestations of depression. When using MRI and [^18^F]FDOPA PET in patients with depression to evaluate the effects of dopamine in causing psychomotor retardation vs. impulsivity, a study showed decreased uptake of [^18^F]FDOPA, indicating lower dopamine uptake in the left caudate of patients with depression who had psychomotor retardation compared to those who had high impulsivity (50). The reduced dopamine activity likely contributed to the observed symptoms of psychomotor dysfunction in those patients with depression (50). A separate study using [^11^C]flumazenil ([^11^C]-FMZ) tracer showed reduced GABA-A binding in the para-hippocampus and superior temporal lobes of the brain (51). When [^11^C]carfentanil was used to measure and assess the presence of mu-opioid receptors (MORs) in the brain (given its strong affinity as an agonist for MORs), multiple regions, including the amygdala and hippocampus showed less MOR availability in patients with subclinical depression (52).

## The utility of PET in the treatment of depression

### Pharmacotherapy

PET has an essential role in research concerning the pharmacodynamics of depression therapies, as it allows for the assessment of drug doses and therapeutic effects based on receptor occupancy. Although earlier studies showed limited ability to discern changes in molecular imaging markers, more recent meta-analysis support the theory of decreased availability of serotonin transporter in depression (53). SSRIs are the most common first-line major depressive disorder medications that target the serotonin transporter. Serotonin transporter blockage in the synaptic cleft is presumed to be the primary mechanism of action in SSRIs. A number of SSRIs have been studied for their ability to occupy and block serotonin transporters (i.e. occupancy) by using PET modalities (47, 54, 55). Occupancy metrics and plasma concentrations can be incorporated into a saturable binding model to estimate a drug’s efficacy (e.g. IC50) and maximum occupancy (e.g. Rmax). Understanding the connection between antidepressant dosage and serotonin transporter occupancy, as well as how different antidepressants interact with serotonin transporters, may help explain why the antidepressant treatment approaches may have variable efficacy. Multiple studies have established a clinical threshold of between 70-80% serotonin transporter occupancy for antidepressants like SSRIs (specifically paroxetine, sertraline, fluvoxamine, citalopram, and fluoxetine), SNRIs, and TCAs (56–59). Serotonin transporter occupancy was higher than norepinephrine transporter occupancy for the same dose of duloxetine (an SNRI) in one study (60); however, the serotonin transporter and norepinephrine transporter occupancy were similar for milnacipran (another SNRI) (61). An [^18^F]FMeNER-D2 PET study showed the antidepressant efficacy of venlafaxine is due to the blockade of the norepinephrine transporter, in addition to the serotonin transporter. The [^18^F]FMeNER-D2 PET study aiming to quantify the binding potential of norepinephrine transporter in the brain of 12 patients with depression found that patients on a daily dose of 150 to 300 mg of venlafaxine had significantly lower binding potential of norepinephrine transporter compared to 9 control subjects. This indicates that clinically relevant doses of venlafaxine extended release block the norepinephrine transporter in the brain of patients with depression. Norepinephrine transporter occupancy varied between 8-61% and increased with the dose, though no clear difference was noted beyond 150 mg/day (62). Relatedly, one [^11^C]DASB PET study found that a 100mg dose of tramadol, an opioid analgesic, corresponded to a 50% occupancy of the serotonin transporter (63).

### Monitoring of pharmacological treatment response

PET has also been used to monitor treatment responses. Multiple cortical brain areas and the midbrain tend to have higher metabolism rates when patients are taking SSRIs (25, 64). Also, when depressed patients on SSRI medications start to feel better, there is often an increase in the number of specific receptors, called 5-HT_2A_, in the frontal cortex of the brain (65, 66). Furthermore, one [^18^F]FDG PET study showed olanzapine (an atypical antipsychotic) and fluoxetine (an SSRI) combination therapy yielded metabolic changes in the brain in patients with therapy-resistant depression, resembling those observed in individuals with therapy-responsive major depression (67).

**Figure 2:**
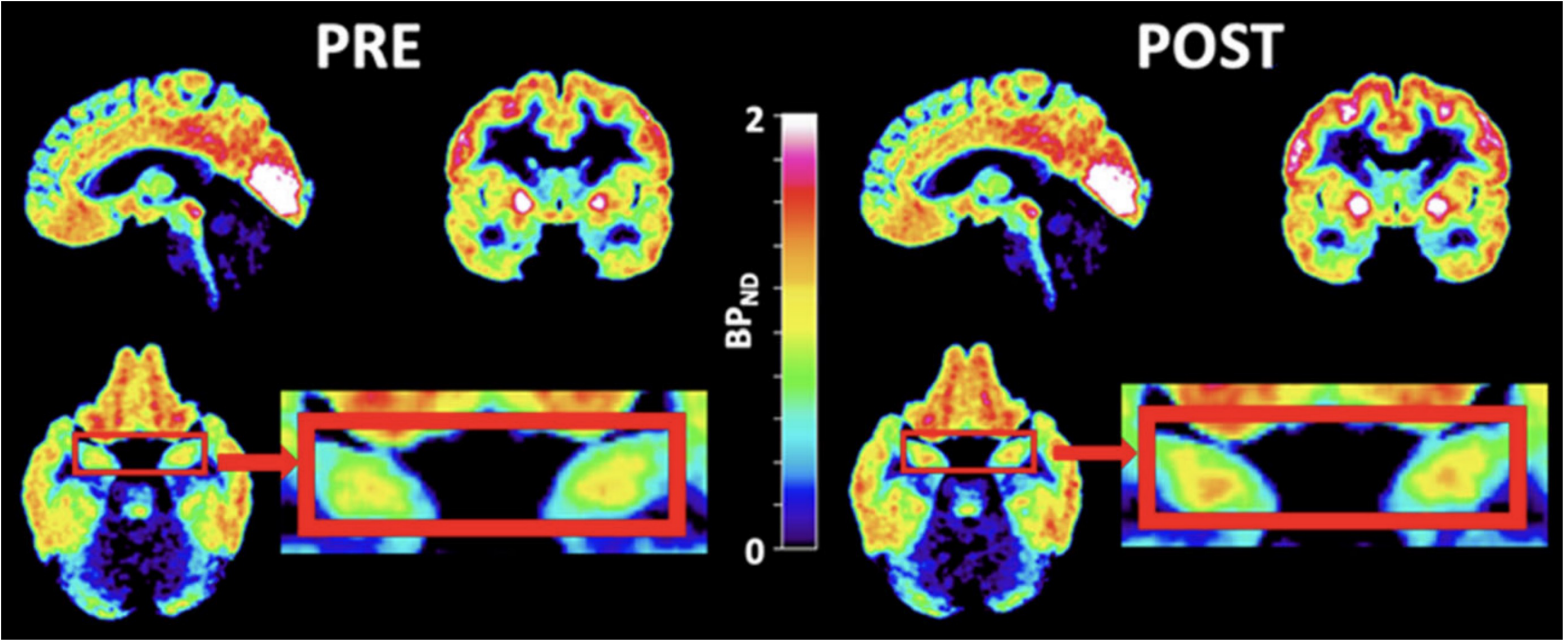
PET images of ketamine-treated patients. PET images of patients zooming in on the hippocampi (red boxes) before (left) and after (right) ketamine treatment. [^11^C]AZ10419369 radiotracer was used in this study. During PET imaging analysis, the regional binding potential for non-displaceable binding (BP_ND_) was calculated using the simplified reference tissue model (SRTM) (74), with the cerebellum as the reference region. [with permission from reference (73)].

Ketamine, an NMDA receptor antagonist, has been suggested to have a possible effective antidepressant effect in acute and chronic settings, though transient issues with cognition, dissociation, amnesia, and perception may exist (68). Ketamine exerts its effect through glutamatergic neurotransmission. There have been mixed findings on the level of glutamate in depression. While a 2019 meta-analysis investigating the level of glutamatergic neuro-metabolite in depression using proton magnetic resonance spectroscopy revealed their decreased levels (69), a 2018 meta-analysis had found no significant differences between the levels of glutamate (70). A study utilizing magnetic resonance spectroscopy to examine whether a ketamine infusion would raise cortical glutamine levels in healthy participants came to the conclusion that slow, low-dose ketamine infusions at antidepressant dosages do not alter cortical glutamate or glutamine in healthy volunteers (71). Similarly, other research implicated the clinical effect of ketamine on depression with a significant reduction in the regulation of G-protein signaling (RGS4), hence inhibiting glutamatergic transmission (72). Although the antidepressant role of ketamine is due to its effect on the glutamatergic system, curiosity exists on how it affects 5-HT_1B_ receptors - which have a more established role in depression. Recently, the effect of ketamine on 5-HT_1B_ receptors in regulating the antidepressant effects has been investigated (73). A [^11^C]AZ10419369 PET study, following a randomized, double-blind, placebo-controlled design, investigated the binding of 5-HT_1B_ receptors before and 24–72 hours after ketamine infusion in SSRI-resistant MDD patients (see **Figure 2**). The study showed that ketamine reduced depression symptoms in MDD patients in a manner inversely related to the binding of the 5HT_1B_ receptor in the ventral striatum at baseline, indicating that 5HT_1B_ receptors could serve as a possible biomarker for monitoring the treatment response of ketamine in depression (73).

### Deep brain stimulation

Deep brain stimulation (DBS) therapy is a safe and effective alternative treatment option for depression that does not respond to other treatments (75). DBS is a procedure in which electrical stimulation of dysfunctional brain circuits leads to focal modulation of their function. On a metabolic basis, DBS leads to increased blood flow and, hence, increased glucose metabolism in the corresponding brain regions. This activates the reward system, heightens the sense of well-being, and decreases symptoms of depression (76). DBS of the subgenual cingulate (Brodmann region 25) has been shown to interrupt focal abnormal activity in limbic-cortical circuits and can significantly reduce symptoms in patients with otherwise therapy-resistant depression (77). [^18^F]FDG PET studies on DBS have corroborated its safety and effectiveness while bolstering evidence concerning mechanistic underpinnings. DBS of the superolateral branch of the medial forebrain bundle has been performed safely and with a rapid antidepressant effect (78).

### Electroconvulsive therapy

**Figure 3:**
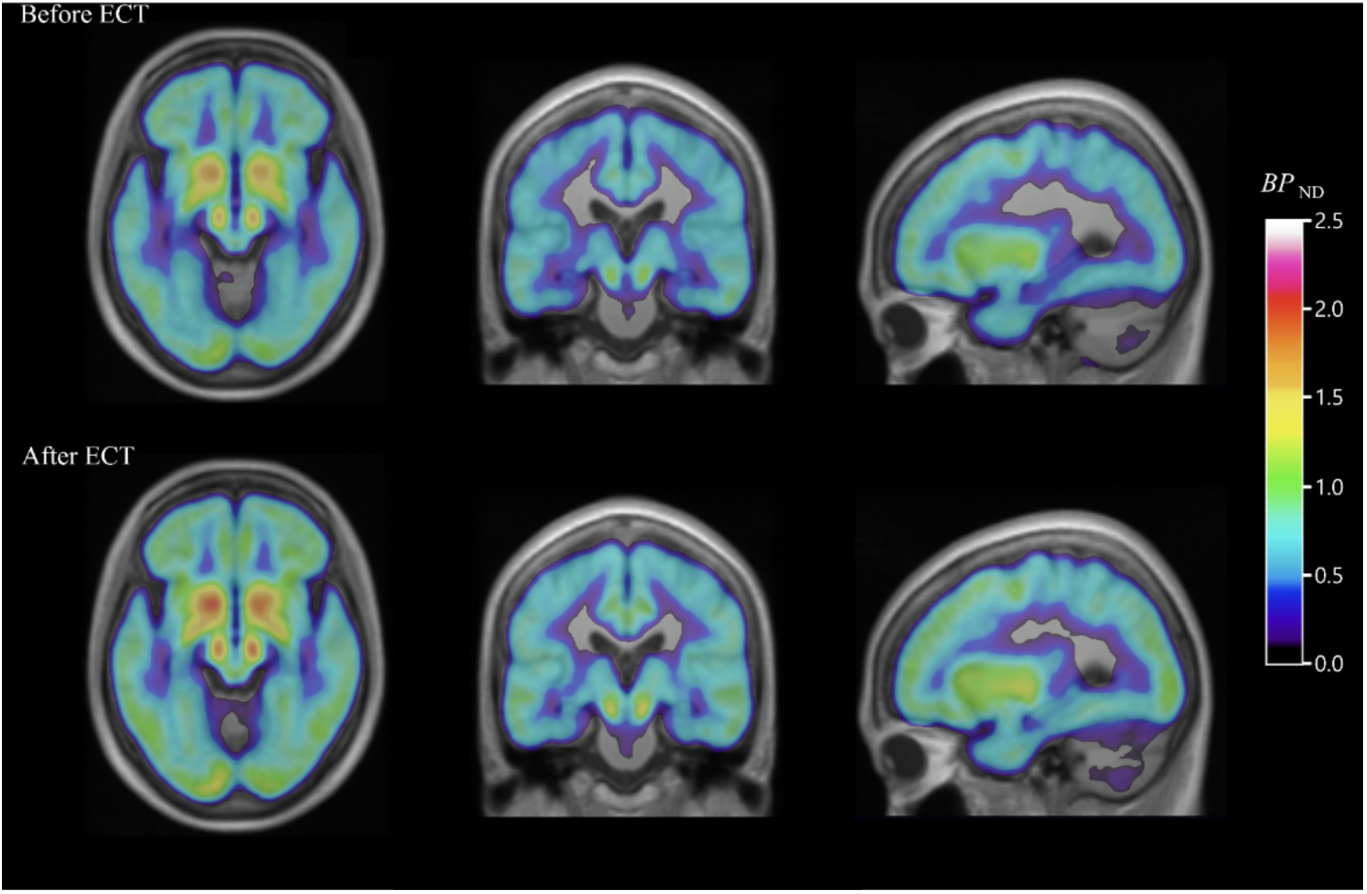
PET images of the serotonin_1B_ receptor effect of electroconvulsive therapy for severe major depressive episodes. Average parametric [^11^C]AZ10419369 PET images overlaid over MR images, showing decreased 5-HT_1B_ receptor binding in the hippocampus before ECT (top) when compared with images taken within one week of (bottom) ECT in major depressive disorder patients [with permission from reference (81)].

Electroconvulsive therapy is another important treatment modality for depression resistant to first-line therapy. Evidence from [^18^F]FDG PET studies suggests that ECT can gradually normalize severe brain hypometabolism in a major depressive episode (78, 79). Other conclusions tentatively drawn from [^18^F]FDG PET studies suggest that ECT improves clinical symptoms by modulating corticolimbic function by increasing limbic/paralimbic metabolism and decreasing neocortical metabolism (80). ECT has also been associated with increased binding of the 5HT_1B_ receptor in the hippocampus of depressed patients following treatment, a mechanism analogous to the clinical improvement observed in patients with treatment-resistant depression after rapid-acting ketamine (**Figure 3**) (81). While the complete mechanism of how electroconvulsive therapy causes improvement of symptoms in depression is precisely not clear, PET imaging could be an essential part of future research that aims to elucidate these pathways. [^18^F]FE-PE2I PET study in depression revealed that ECT reduces dopamine transporter binding in the striatum. This decrease was associated with a reduction in the severity of depression, as measured by standard rating scales. The results of this research suggest that alterations in the brain’s dopamine system may contribute to the antidepressant effects of electroconvulsive therapy (82). Using [^11^C]FLB 457 PET, another brain imaging study supported the idea that ECT for depression possibly works through a dopamine-related mechanism. In this study, all 7 patients with depression responded to ECT, and they had reductions in the binding of dopamine D2 receptors in the rostral anterior cingulate region of the brain after the treatment as compared to the measurements before ECT (83). Similarly, a different PET study using [^11^C]raclopride (a selective dopamine D2/D3 antagonist tracer) found patients experiencing major depressive episodes had lower dopamine D2/D3 binding in the striatum compared to those who did not have depression. However, in this study, ECT did not result in a significant improvement in D2/D3 binding in patients with depression (84).

### Alternate therapies

Given the subjective interpretation and self-perception of mental activities that affect neural circuitry and plasticity at multiple levels of functioning, mentalistic elements must be considered alongside psychotherapy for successful depression treatment (85). PET imaging has furthered our understanding of the neural changes associated with alternative treatment modalities and psychotherapies.

Neuroinflammation has been implicated in the pathophysiology of depression. Since microglia are crucial to neuroinflammation, Li et al. conducted a PET study using [^18^F]FEPPA to investigate the translocator protein total distribution volume (TSPO V_T_). TSPO is a surrogate of the activity of microglia and, hence, neuroinflammation and TSPO V_T_ is an index of TSPO density. According to this [^18^F]FEPPA-PET study, the microglial density in individuals with major depression was reduced in the neocortex gray matter, temporal cortex, frontal cortex, and hippocampal regions following clinical improvement after cognitive behavioral therapy (CBT) treatment, therein bolstering evidence supporting the efficacy of CBT for depression (86).

## Conclusion

PET is becoming increasingly viable as an imaging tool for diagnosis, treatment, and management of depression. PET imaging has shown useful findings in depression with respect to differences in metabolism in the brain, involved neurotransmitters, neuroreceptors, the disease’s severity and duration, the pharmacodynamics of different antidepressants, and the neurobiological underpinnings of non-pharmacological treatments such as psychotherapy, electroconvulsive therapy, and deep brain stimulation therapy. Although many studies related to [^18^F]FDG and [^15^O]-water have found some kind of metabolic dysfunction, they do not agree on which parts of the brain are implicated. This is true for amyloid PET as well. The possible reason could be heterogeneity among the studies and the small sample size. The absence of uniform findings in this context has prevented the clinical translation of [^18^F]FDG, [^15^O]-water, and amyloid PET in depression. Several neurotransmitter-related PET findings have been associated with clinical manifestations of depression, the important ones being decreased 5-HT_1A_ receptors in several parts of the brain. However, the determination of 5-HT_1A_ in depression has been confounded by quantification technique.

PET studies have indicated disruptions in various other neurotransmitter systems (such as dopamine, GABA, and glutamate) as underlying pathophysiology of depression. PET has demonstrated reductions in dopamine uptake, decreased GABA-A receptor binding, and altered excitatory neurotransmission, particularly involving glutamate. PET scans with the [^11^C]ABP688 tracer have consistently shown lower mGluR5 receptor binding in depressed patients, a finding that correlates with the severity of depression (associated with mGluR5 binding in the hippocampus) and is supported by western blot analyses of postmortem brain samples. This highlights the potential for these neurochemical markers to inform both the diagnosis and treatment of depressive disorders.

There is limited research on the neuropathological mechanisms underlying various clinical signs of depression (e.g., catatonia, psychosis, negative symptoms).

PET imaging has been pivotal in studying the pharmacodynamics of antidepressants, with the potential to reveal a clinical threshold for serotonin transporter occupancy necessary for the effectiveness of common antidepressants. PET can also reveal neurotransmitter effects that, in fact, are not the primary target of the treatment. It has been instrumental in uncovering the effects of medications like duloxetine, venlafaxine, and milnacipran on neurotransmitter transporters and highlighting the relation between the dose of tramadol and serotonin transporter occupancy. Similarly, PET has been used to monitor metabolic and receptor-level changes in response to antidepressants. It has shown metabolic changes in brain regions due to SSRI treatment and has associated clinical improvements due to SSRI with rises in 5-HT_2A_ receptor numbers in the frontal cortex. Similarly, 5-HT_1B_ is affected in depression and possibly influenced by treatments as different as CBT, ECT, and ketamine. The utility of PET in understanding the efficacy of treatments like olanzapine and fluoxetine combination therapy and in discerning the effects of ketamine infusion on 5-HT_1B_ receptor binding is underscored. PET has provided evidence regarding the safety and effectiveness of DBS in depression and elucidated its impact on neural metabolism and connectivity. While the exact mechanism of ECT remains to be fully understood, reduction in dopamine transporter binding in the striatum has been proposed as a contributing factor to its antidepressant effects. Besides, PET imaging has shed light on the neural changes associated with alternative therapies and psychotherapies, such as sleep limitation therapy and CBT. PET has highlighted the changes in glucose metabolism after sleep limitation therapy, reduction in neuroinflammation post-CBT, and the role of serotonin and its 5-HT_1B_ receptor in the physiological response psychotherapy. PET imaging may be a crucial tool in the ongoing quest to elucidate the complex biological mechanisms of depression and its treatment, offering valuable insights that have the potential to guide more effective, personalized interventions for this disorder. So far, the research findings have not yet resulted in significant changes in clinical practice, for example, in regular diagnosis and work-up of depression.

## Future considerations

There are many instances of conflicting results obtained in depression despite a similar PET imaging approach applied in different studies. This may require identification of the underlying heterogeneity and possibly standardization of quantification technique in large sample size studies. Additional studies targeting patient subpopulations across both mental and physical health domains need to be carried out. Similarly, more basic science research is necessary to further elucidate the genetic basis of depression and the neurobiological mechanisms of catatonia, bipolar disorder, and associated psychosis. For instance, continued research into various neurotransmitters (e.g., dopamine, glutamate, serotonin, norepinephrine) and their associated receptors and transporters would lend insight into the heterogeneous manifestations of depression and allow for the development of more targeted treatment strategies. Additionally, more research is required to determine the biological underpinnings of the treatment response to psychotherapies, ECT, DBS, and other alternative therapies. As such, given the increasing role of molecular imaging in drug development and precision medicine initiatives, PET can contribute significantly to more timely screening, diagnosis, and management of depression in the near future.

## Author contributions

SS: Investigation, Writing – original draft. AT: Investigation, Writing – original draft. MK: Writing – original draft. RK: Writing – original draft. CA: Writing – original draft. KP: Writing – original draft. YB: Writing – original draft. TW: Writing – review & editing. AA: Writing – review & editing. M-ER: Supervision, Conceptualization, Writing – review & editing.
